# Screen Identifying *Arabidopsis* Transcription Factors Involved in the Response to 9-Lipoxygenase-Derived Oxylipins

**DOI:** 10.1371/journal.pone.0153216

**Published:** 2016-04-13

**Authors:** Elisabeth Walper, Christoph Weiste, Martin J. Mueller, Mats Hamberg, Wolfgang Dröge-Laser

**Affiliations:** 1 Julius-von-Sachs-Institute, University of Würzburg, Julius-von-Sachs-Platz 2, D-97082 Würzburg, Germany; 2 Division of Physiological Chemistry II, Department of Medical Biochemistry and Biophysics, Karolinska Institute, S-171 77 Stockholm, Sweden; Institute of Genetics and Developmental Biology, Chinese Academy of Sciences, CHINA

## Abstract

13-Lipoxygenase-derived oxylipins, such as jasmonates act as potent signaling molecules in plants. Although experimental evidence supports the impact of oxylipins generated by the 9-Lipoxygenase (9-LOX) pathway in root development and pathogen defense, their signaling function in plants remains largely elusive. Based on the root growth inhibiting properties of the 9-LOX-oxylipin 9-HOT (9-hydroxy-10,12,15-octadecatrienoic acid), we established a screening approach aiming at identifying transcription factors (TFs) involved in signaling and/or metabolism of this oxylipin. Making use of the *At*TORF-Ex (*A**rabidopsis* *t**haliana* Transcription Factor Open Reading Frame Expression) collection of plant lines overexpressing TF genes, we screened for those TFs which restore root growth on 9-HOT. Out of 6,000 lines, eight TFs were recovered at least three times and were therefore selected for detailed analysis. Overexpression of the basic leucine Zipper (bZIP) TF TGA5 and its target, the monoxygenase CYP81D11 reduced the effect of added 9-HOT, presumably due to activation of a detoxification pathway. The highly related ETHYLENE RESPONSE FACTORs ERF106 and ERF107 induce a broad detoxification response towards 9-LOX-oxylipins and xenobiotic compounds. From a set of 18 related group S-bZIP factors isolated in the screen, bZIP11 is known to participate in auxin-mediated root growth and may connect oxylipins to root meristem function. The TF candidates isolated in this screen provide starting points for further attempts to dissect putative signaling pathways involving 9-LOX-derived oxylipins.

## Introduction

Membrane oxidation by Reactive Oxygen Species (ROS) is harmful to cell integrity and survival but is also a consequence of life in an oxygen-containing atmosphere. Lipid peroxidation yields a plethora of oxidized polyunsaturated fatty acids (FA), summarized as oxylipins [1,2]. In animals, oxylipins such as prostaglandins or leukotrienes are well-known signaling molecules [3]. Some oxylipins, in particular highly reactive α,ß-unsaturated carbonyl compounds (Reactive Electrophilic Species; RES), display toxic and/or mutagenic properties [4]. On the other hand, low levels of RES have been well defined as signaling molecules in animals, triggering gene expression of protective pathways [1,3].

In plants, oxylipins are formed non-enzymatically or by enzymatic activities [1,4]. Enzymatic oxygenation of linoleic acid (18:2) and linolenic acid (18:3) by LIPOXYGENASES (LOXs) or α-dioxygenases (α-DOXs) results in highly reactive hydroperoxides (FAOOHs) which are further converted into various oxylipin species [5,6]. According to their substrate regiospecificity with respect to the carbon oxygenated, 9-LOX and 13-LOX enzymes are distinguished. In *Arabidopsis* six *LOX* gene family members have been described, encoding 9-LOXes (LOX1, LOX5) and 13-LOXes (LOX2, 3, 4, 6) [6]. Whereas keto-FAs are probably generated by LOX activity from FAs, hydroxy-FAs are derived from LOX-generated FA peroxides via yet not clearly assigned reductases. However, accumulating evidence suggests that caleosin/peroxygenases may perform this reductase activity [7,8].

With respect to the plant pro-hormone Jasmonic Acid (JA), biosynthesis and signaling are well-described [9,10]. Perception as an isoleucine conjugate (JA-Ile) by the COI-1 (CORONATIN INSENSITIVE1) F-protein, proteasome dependent degradation of JAZ-type (JASMONATE-ZIM DOMAIN) repressors and gene activation via MYC2-related bHLH (BASIC-HELIX-LOOP-HELIX) transcription factors (TFs) clearly defines this 13-LOX-derived oxylipin as a potent signaling molecule in various aspects of development and stress defense [9]. By screening of *Arabidopsis* seedlings using various oxylipins with respect to root growth and their dependence on COI-1, at least three independent signaling pathways for oxylipins have been postulated [11]. Whereas jasmonates and cyclopentenones elicit a COI-1-dependent overall arrest of root growth, short chain ω-oxoacids and divinyl ethers lead to growth arrest with loss of apical dominance. The third group of keto- and hydroxy-FA induce root waving in a COI-1-independent manner.

In contrast to JA, our knowledge on the signaling of RES oxylipins in plants is limited. RES compounds such as the cyclopentenone OPDA (12-Oxo-Phytodienoic Acid) or phytoprostane A1 (PPA1) have been shown to inhibit cell cycle progression and root growth [12]. Moreover, transcriptome analysis revealed that these compounds activate gene expression distinct from the JA response, particularly related to detoxification, stress response and secondary metabolism. The use of a triple mutant impaired in three *Arabidopsis* bZIP factor genes (*tga2 tga5 tga6*) [13] revealed that these TFs are controlling a substantial fraction of this oxylipin-induced gene expression [12]. Moreover, class 2 TGAs recruit the transcriptional co-activator SCARECROW-LIKE 14 (SCL14) to control detoxification responses to various endogenous and xenobiotic compounds [14]. One well-defined target gene encodes a CYTOCHROME P450 monoxygenase enzyme (CYP81D11) which is probably involved in the detoxification response [15]. However, the substrates are currently not identified.

During the last years, evidence supporting a signaling function of 9-LOX-oxylipins in plant development and stress response has accumulated. When applied extracellularly, 9-HOT (9-hydroxy-10,12,15-octadecatrienoic acid) and 9-KOT (9-keto-10,12,15-octadecatrienoic acid) reduce lateral root initiation. In contrast, genetic approaches using a 9-LOX specific *lox1/lox5* double mutant display an increased number of lateral root primordia [11]. 9-HOT has been shown to induce a root growth response (“waving”) which was proposed to be due to localized callose deposition. 9-HOT also increases tolerance to ROS and enhances pathogen defenses [11,16–19]. Indeed, 9- and 13-ketodienes have been detected in *Arabidopsis* leaves upon *Pseudomonas syringae* infection [20]. Recently, 10-OPEA (10-oxo-11-phytoenoic acid) a 9-LOX-derived compound analogous to OPDA, has been identified to play an important role in fungal defense in maize [21].

Supporting evidence that 9-LOX-oxylipins perform as signaling molecules comes from genetic approaches identifying *Arabidopsis* mutants in the response to 9-HOT (“*noxy*” mutants, NON-RESPONDING TO OXYLIPINS) [11]. The response to 9-HOT is independent of JA signaling, as it remains active in a *coi-1* mutant. Some *noxy* mutants are allelic to the ethylene signaling mutants *ctr1* (CONSTITUTIVE ETHYLENE RESPONSE1) and *eto1* (ETHYLENE-OVERPRODUCER1) and accordingly, 9-HOT and ethylene were found to act antagonistically [19]. Nevertheless, no perception system has been described, yet.

In order to get an insight into putative 9-LOX-oxylipin signaling pathways in plants, we set-up a screen aiming at TFs involved in the transcriptional response to 9-HOT. The *At*TORF-EX (*A**rabidopsis* *t**haliana* Transcription factor Open Reading Frame Expression) collection holds seeds of transgenic TF overexpressing plants, which can be used for identifying regulators in development or stress response [22,23]. Using GATEWAY^®^ technology, a batch transformation procedure has been developed, which results in seed pools derived from transformants of 30–50 TF genes under control of the Cauliflower 35S promoter (35S). Currently, this screening collection enables analysis of more than 700 TFs and has successfully been applied for several screening purposes [23,24]. Here, we identify and re-evaluate a set of TFs which when overexpressed result in tolerance to 9-HOT.

## Materials and Methods

### Plants lines and culture

For root growth assays, *Arabidopsis* Columbia (*Col-0*) wt and *At*TORF-Ex seeds [22,23] were sterilized, stratified and germinated on MS medium [25] under long-day conditions. After 5d, seedlings were transferred to MS medium supplemented with 25μM 9-HOT. On day 10, plates were scanned and root length was measured using the *Roots tool* software (zysko@tlen.pl). To study gene induction by flooding, 2-week old seedlings were grown on MS medium and submerged with water for 1–6h. For chemically-induced gene expression, 10 μM Estradiol (Sigma, Munich, Germany) was added.

The following mutant lines were used: *tga2*,*5*,*6*, *tag2*,*5*, *tag6* [13], the complementation lines *tga2*,*5*,*6 TGA5g* [13] and *tga2*,*5*,*6* (35S:TGA2) and *tga2*,*5*,*6* (35S:TGA5) [26], 35S:CYP81D11 and *cyp81d11* [27]. The Est-inducible lines XVE-bZIP11 and XVE-amibZIP2,-11,-44 are described in [28,29]. T-DNA insertion lines *erf106* (SALK_097771) and *erf107* (SALK_015182) were obtained from the Nottingham Arabidopsis Stock Centre and crossed for generating *erf106/erf107* double mutants.

### Chemical treatments

The following compounds were tested in root growth assays using the indicated concentrations. 9-HOT (9(*S*)-hydroxy-10(*E*),12(*Z*),15(*Z*)-octadecatrienoic acid), 9-KOT (9-keto-10(*E*),12(*Z*),15(*Z*)-octadecatrienoic acid) and OPDA (12-Oxo-Phytodienoic Acid) were prepared as described in [11,12], respectively. TIBA (2,3,5-triiodobenzoic acid), Naphthalene-Acetic Acid (NAA) and Jasmonic Acid (JA) were obtained from Sigma (Munich, Germany).

### Generation of transgenic plants

*ERF106* (At5g07580) and ERF107 (At5g61590) orfs were PCR-amplified using the primers provided in S1 Table and introduced into the entry vector pDONR201 (Invitrogen, Germany) and the XVE [28] expression vector pMDC7-N’HA [28,29] by BP- or LR-reaction, respectively. These binary vectors enable expression of N-terminal HA fusion proteins in plants. Floral dip transformations have been performed as described in [30] using the *Agrobacterium tumefaciens* strain GV3101.

### Molecular Biology Methods

To identify TF genes in the selected *At*TORF-Ex lines, PCR-amplification was performed as described in [22] using primers given in S1 Table. Quantitative Reverse-Transcriptase PCR (RT-qPCR) was carried out according to [31] using SYBR green detection (Sigma, Munich, Germany) and the primers given in S1 Table. Cycling conditions were as follows: 5 min at 98°C, 36 cycles of 30 s at 95°C, 45 s at 55°C and 60 s at 72°C, linked to a default dissociation stage program to detect non-specific amplification. Calculations were performed according to the 2^–ΔCT^ method [32] using the *UBQ5* (At3g62250) gene as a reference. Immunoblots were performed as described in [31] using α-HA antibodies (ChIPgrade) from rabbit (1:2000 dilution) (abcam, Cambridge, UK).

**Bioinformatic and statistical analyses** were performed with the Excel GraphPad Prism, Origin and XCELL software using the statistic tests indicated in the figure legend.

## Results and Discussion

### Screening for transcription factors involved in oxylipin signaling and detoxification

**Fig 1:**
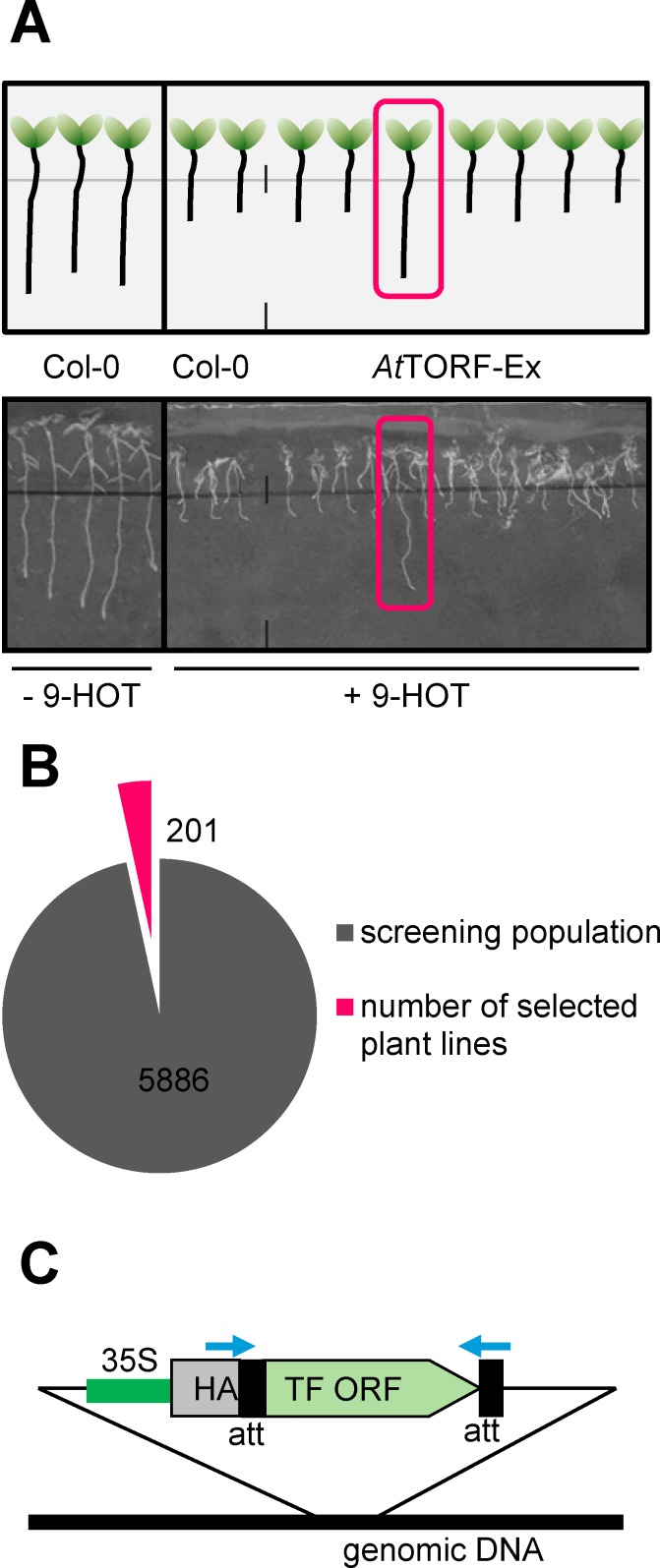
Schematic overview of the *At*TORF-Ex screening. (A) Col-0 and *At*TORF-Ex [21] seedlings are cultivated on MS-Medium without or with 25μM 9-HOT for 5 d. Individuals showing unimpaired root growth on the 9-LOX-oxylipin (marked in red) were isolated for further analysis. (B) Overview of screening results: Out of 6000 *At*TORF-Ex lines, 201 individuals were selected. (C) *At*TORF-Ex transgenes are flanked by GATEWAY^®^ att-sites (black) which enable PCR-amplification and sequencing of TF genes using the indicated primers (blue arrows). 80 independent TFs were identified and listed in Table 1. Due to N-terminal fusion of HA-tag, TF genes encoded proteins can be detected via immunoblot.

Aiming at TF function involved in the responses to 9-LOX-oxylipins, we assayed the root growth of 2-week-old *Arabidopsis* seedlings on MS-Agar supplemented with 25 μM 9-HOT. In contrast to [11], a “wavy” root growth phenotype was not reproducibly observed under our experimental conditions, but root apical growth was severely impaired (Fig 1A). Based on the hypothesis that oxylipin signaling or detoxification programs are transcriptionally controlled, a screen based on overexpression of TFs should identify crucial regulators involved in these processes. Here, we established a screen using the *At*TORF-Ex resource [22], a collection of GATEWAY^®^-derived *Arabidopsis* TF overexpression lines employing the 35S promoter and scored plants showing wildtype (wt) root growth after 9-HOT application to the media. Screening of more than 6000 seedlings recovered 201 individuals (3%) which display tolerance to 9-HOT (Fig 1B). According to the calculations in [22], the sample size should be statistically sufficient to cover all possible TFs in the screen with a probability of 99%. Due to the conserved GATEWAY^®^-att sequences flanking the TF orfs in the *At*TORF-Ex transgenic lines, the respective overexpressed TF genes can easily be recovered by PCR (Fig 1C). As summarized in Table 1, several TF genes have been isolated up to 5 times, and hence their overexpression leads reproducibly to 9-HOT tolerant root growth (e.g. ERF106, ERF107, MADS-box factor AGL86). Members of the bZIP family are clearly overrepresented among the TFs leading to 9-HOT tolerance. This is particularly interesting, as bZIP members have been described as regulators of protective pathways towards endogenous or exogenous oxylipin molecules in plant and non-plant species [12,14,15,33–35]. However, evolutionary conservation is not supported by homology outside the bZIP domain. Frequently, we observed that expression of related family members resulted in the same growth phenotype. Presumably, these TFs share related binding properties and functional redundancies. This was particularly true for bZIP factors of the group S [36], which overall were found 18 times in the screen. The most abundant bZIP members are the highly related TFs bZIP11 and bZIP2 (overall 7 times). Furthermore, CRFs are a group of TFs which modulate cytokinine signaling [37,38]. Although single CRFs were found only twice, overall 3 related group members were detected. Cytokinine has a crucial function in controlling root meristem size [39] and hence, CRF overexpression may overcome the block in root growth caused by 9-HOT. In contrast, TFs identified only once or twice are less likely candidates. Nevertheless, less abundant TFs may also be important but might have missed for technical reasons. We have selected several TF candidates to study their impact in the tolerance mechanism to 9-HOT in more detail.

**Table 1:** Overview of the screening results. TF transgenes were PCR-amplified from those *At*TORF-Ex lines which display restored root growth on 9-HOT. Frequency: TFs found 3–5 time were given in bold.

At identifyer	frequency	TF family, subgroup	TF name
**AT4G34590**	**4x**	**bZIP, group S**	**bZIP 11, ATB2**
**AT2G18160**	**3x**	**bZIP, group S**	**bZIP2, GBF5**
**AT1G68880**	**3x**	**bZIP, group S**	**bZIP8**
**AT5G49450**	**2x**	bZIP, group S	bZIP1
**AT5G60830**	**2x**	bZIP, group S	bZIP70
AT1G59530	1x	bZIP, group S	bZIP4
AT2G04038	1x	bZIP, group S	bZIP48
AT3G30530	1x	bZIP, group S	bZIP42
AT3G49760	1x	bZIP, group S	bZIP5
**AT5G24800**	**2x**	bZIP, group C	bZIP9
AT5G28770	1x	bZIP, group C	bZIP63
**AT4G36730**	**3x**	**bZIP, group G**	**bZIP41, GBF1**
**AT2G35530**	**2x**	bZIP, group G	bZIP16
**AT5G44080**	**2x**	bZIP, group A	bZIP13
**AT4G34000**	**2x**	bZIP, group A	ABF3
AT2G36270	1x	bZIP, group A	ABI5
**AT1G06070**	**2x**	bZIP group I	bZIP69
AT1G43700	1x	bZIP, group I	bZIP51, VIP1
AT2G40620	1x	bZIP, group I	bZIP18
**AT1G42990**	**2x**	bZIP	bZIP60
AT5G06950	1x	bZIP, group D	bZIP20, TGA2
AT5G06960	1x	bZIP, group D	bZIP26, TGA5
AT1G22070	1x	bZIP, group D	bZIP22, TGA3
AT1G77920	1x	bZIP, group D	bZIP50, TGA7
AT1G08320	1x	bZIP group D	bZIP21, TGA9
AT2G16210	1x	B3 family	
**AT4G25400**	**2x**	bHLH, group Ib	bHLH118
**AT2G42300**	**2x**	bHLH, group XII	bHLH048
AT5G62610	1x	bHLH, group XII	bHLH079
**AT3G06590**	**2x**	bHLH	
**AT5G15160**	**2x**	bHLH	BNQ2
AT1G09250	1x	bHLH	
AT3G17100	1x	bHLH	
AT4G28140	1x	ERF, group I	ERF054
AT5G65130	1x	ERF, group I	ERF057
AT4G06746	1x	ERF, group II	ERF007, RAP2.9
**AT2G23340**	**2x**	ERF, group II	ERF008, DEAR3
**AT4G36900**	**2x**	ERF, group II	ERF009, Rap2.10
AT1G77640	1x	ERF, group II	ERF013
AT1G22810	1x	ERF, group II	ERF019
**AT1G01250**	**2x**	ERF, group III	ERF023
AT3G60490	1x	ERF, group III	ERF035
AT4G32800	1x	ERF, group III	ERF043
**AT5G53290**	**2x**	ERF, group VI	ERF065, CRF3
**AT4G27950**	**2x**	ERF, group VI	ERF066, CRF4
AT1G22985	1x	ERF, group VI	ERF069, CRF7
AT3G16770	1x	ERF, group VII	ERF072, Rap2.3
AT1G72360	1x	ERF, group VII	ERF073
AT1G06160	1x	ERF, group IX	ERF094, ORA59
**AT5G43410**	**2x**	ERF, group IX	ERF096
AT1G04370	1x	ERF, group IX	ERF097, ERF14
AT3G23230	1x	ERF, group IX	ERF098, TDR1
AT4G17500	1x	ERF, group IX	ERF100, ERF1
AT5G47220	1x	ERF, group IX	ERF101, ERF2
AT5G61600	1x	ERF, group IX	ERF104
AT5G51190	1x	ERF, group IX	ERF105
**AT5G07580**	**5x**	**ERF, group IX**	**ERF106**
**AT5G61590**	**5x**	**ERF, group IX**	**ERF107**
AT1G28370	1x	ERF, group VIII	ERF076, ERF11
AT1G53170	1x	ERF, group VIII	ERF079, ERF8
AT3G20310	1x	ERF, group VIII	ERF083, ERF7
**AT5G13910**	**2x**	ERF, group VIII	ERF085, LEP
AT1G28160	1x	ERF, group VIII	ERF087
AT1G12890	1x	ERF, group VIII	ERF088
**AT1G12980**	**2x**	ERF, group VIII	ERF089, ESR1
AT4G34410	1x	ERF, group X	ERF109, RRTF1,
**AT4G13040**	**2x**	AP2/ERF soloist	APD1
AT5G04760	1x	Homeodomain-like	
AT5G08520	1x	Homeodomain-like	
AT5G52660	1x	Homeodomain-like	
**AT4G39250**	**2x**	Hsf	RL1
AT3G22830	1x	Hsf	HSFA6B
**AT1G31630**	**5x**	**MADSbox**	**AGL86**
**AT1G48150**	**3x**	**MADSbox**	
AT1G22640	1x	MYB	MYB3
AT1G70000	1x	MYB-like	
**AT1G19040**	**2x**	NAC	
**AT5G50820**	**2x**	NAC	Nac097
AT1G02210	1x	NAC	
AT5G18300	1x	NAC	Nac088
AT1G56280	1x	Zn-Finger C2H2	DI19

### Expression of the bZIP transcription factor TGA5 and its target gene CYP81D11 provoke tolerance to 9-HOT

**Fig 2:**
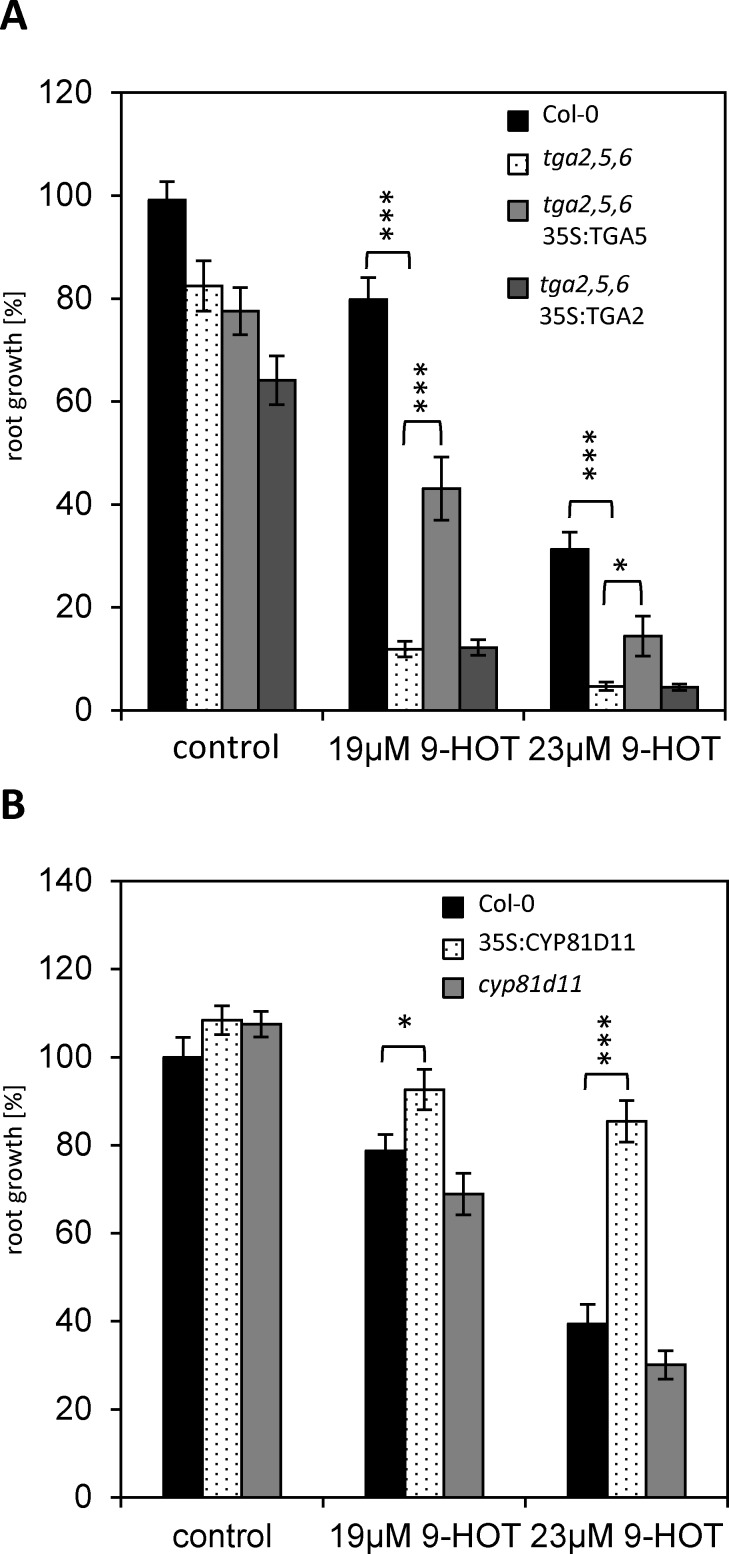
Expression of TGA5 and its target gene *CYP81D11* are mediating tolerance to 9-HOT. Root growth of several *Arabidopsis* transgenic lines has been quantified comparing 9-HOT treated and untreated roots. The untreated wt (Col-0) is set to 100%. Results obtained from the *Arabidopsis* triple TGA mutant *tga2*,*5*,*6* and lines expressing TGA5 or TGA2 under control of the 35S promoter in the *tga2*,*5*,*6* background (A) or 35S:CYP81D11 and *cyp81d11* (B) are shown. Given are mean values +SE (n≥22). The indicated statistical differences are calculated using the student’s *t*-test: *p ≤ 0.05, **p≤0.01, ***p ≤ 0.001.

TGA-bZIPs (group D) [36] have already been demonstrated to be involved in the response to 13-LOX-derived oxylipins as well as lipophilic RES-xenobiotics and therefore may serve as a proof-of-principle that TF overexpression results in tolerance to oxylipins and other lipids [12,14, 15]. Although several TGA members were detected to increase root length on 9-HOT, all these TFs were found only once in our screen. Nevertheless, we studied the impact in response to 9-LOX-oxylipins in more detail. In comparison to wt, the triple mutant of the functionally related TGA factors *tga2*,*tga5*,*tga6* (*tga2*,*5*,*6*) is strongly impaired in primary root growth on medium containing 19μM 9-HOT (Fig 2A). Although these TFs have been described as redundant [13] we assayed single *tga6* and *tga2*,*5* double mutants (S1 Fig). Whereas, *tga2*,*5* shows an intermediate responses with respect to wt and triple mutant, root growth of *tga6* mutants was comparable to wt (S1 Fig). Moreover, complementation of *tga2*,*5*,*6* with a genomic *TGA5* fragment (*TGA5g*) (S1 Fig) or 35S-driven expression of TGA5 (Fig 2) could partially revert the mutant phenotype. This is not the case for 35S-driven expression of TGA2 in the triple mutant (Fig 2A). Taken together, these data support the view, that distinct differences between the TGA members can be seen, as it was recently described in [40]. Nevertheless, these assays do not reveal the quantitative contribution of each TGA member. In agreement with previous findings [14,15], this data supports the view, that TGAs control a wide spectrum of responses to support growth on 9- and 13-LOX-derived oxylipins and xenobiotic compounds. Indeed, genome-wide transcriptome analyses define a broad array of detoxification genes controlled by these TFs [12].

TGAs have been proposed to be involved in controlling detoxification pathways with respect to oxylipins or xenobiotics. The cytochrome P450 monoxygenase gene *CYP81D11* is transcriptionally induced by lipid peroxidation products like phytoprostanes, OPDA and JA as well as electrophilic xenobiotics (e.g. 2,4-dichlorophenoxyacetic acid, benoxacor, or the auxin-transport inhibitor TIBA (2,3,5-triiodobenzoic acid) [14,15]. Along this line, plants constitutively expressing CYP81D11 show significantly increased root growth on 9-HOT containing media (Fig 2B), demonstrating that the CYP81D11 enzyme is sufficient to provide full tolerance to 9-HOT. Hence, although the enzymatic function of CYP81D11 is not well-defined yet, these and previous data propose a function in metabolizing both 9- and 13-LOX-derived oxylipins. However, in comparison to wt, root growth of the *cyp81d11* mutant on 9-HOT is slightly but not significantly reduced. Most likely, other CYP450 proteins may share redundant functions. Further studies are needed to define the substrate specificity of CYP81D11 with respect to xenobiotics and endogenous compounds.

### Down-regulation of bZIP11-related group S bZIP factors increases root growth on 9-HOT

**Fig 3:**
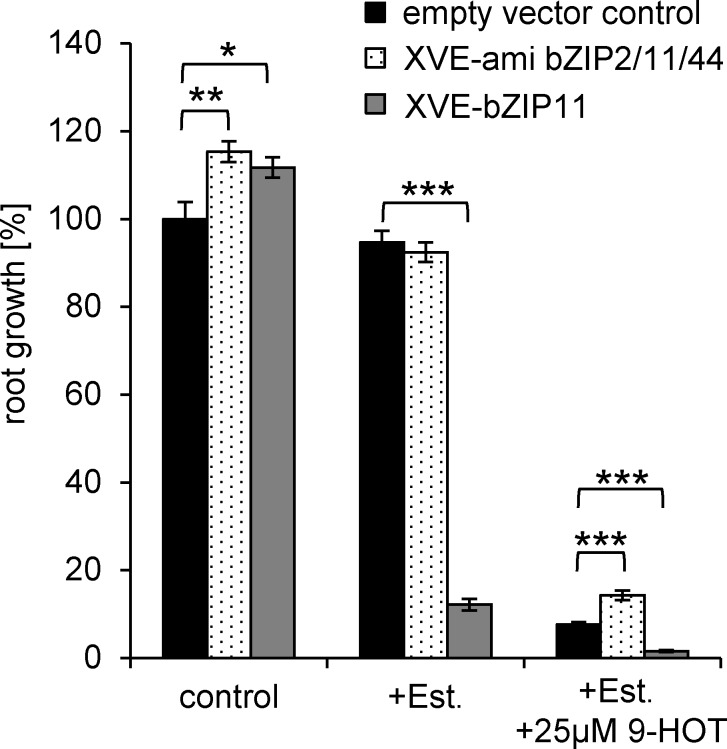
Down-regulation of bZIP11-related TFs enhance root growth upon 9-HOT treatment. Root growth of Est-inducible bZIP11 overexpressing plants (XVE-bZIP11) or artificial micro RNA lines (XVE-amibZIP2/11/44) was quantified after application of Est (10μM) or Est + 9-HOT (25μM). The untreated wt (Col-0) is set to 100%. Given are mean values +SE (n≥63). The indicated differences are calculated using the student’s *t*-test: *p ≤ 0.05, **p≤0.01, ***p ≤ 0.001.

Among the group S bZIP members identified in the *At*TORF-Ex screen, bZIP11 and bZIP2 lines were most frequently found (overall 7 times). This finding is in contrast to previous studies, demonstrating that overexpression of these bZIPs leads to severely reduced plant growth [41]. We confirmed these findings, as Estradiol (Est) inducible expression of bZIP11 (XVE-bZIP11) led to strongly impaired root growth even in the absence of 9-HOT (Fig 3). In agreement with these observations, molecular inspection by RT-qPCR revealed, that the *At*TORF-Ex lines isolated showed reduced bZIP transcript levels (S2 Fig). These findings can be explained by co-suppression events [22]. As no viable bZIP11 T-DNA-insertion lines were found, we applied an Est-inducible artificial microRNA (ami) approach targeting three highly related genes, namely *bZIP11*, *bZIP2* and *bZIP44* (XVE-amibZIP2,11,44) [28]. These three group S bZIPs are highly related and partially redundant TF and hence, have been found several times in our screen. As depicted in Fig 3, 9-HOT impaired root growth can partially, but significantly be reverted by the Est-induced bZIP knock-down. The small increase in root growth might be explained by the fact, that the amiRNA approach reduces target transcript levels to only 20–40% [28]. bZIP11-related bZIPs have been implicated in metabolic adaptation upon energy limiting conditions [41–45]. Moreover, these bZIPs are proposed to function in controlling auxin-mediated transcription and root growth [28 and Weiste unpublished]. Indeed, the bZIP11 heterodimerisation partner bZIP63 (group C), which has also been identified in the screen (Table 1), is directly phosphorylated and controlled by the major integrator of low energy stress, SnRK1 (Snf1 RELATED KINASE1) [43]. Moreover, group G bZIPs (GBF1, *At*bZIP41, *At*bZIP16), which have been shown to heterodimerize with S and C bZIPs [46] were also found in the screen. The underlying mechanism, *i*.*e*., precisely how these bZIPs control root apical growth, is currently not resolved. Particularly, 9-LOX-oxylipins have been implicated in controlling overall root growth and lateral root formation [11] and an impact of oxylipins on cell cycle has been proposed [12]. Here, we demonstrate that 9-HOT interferes with a transcriptional regulatory system which down-regulates root apical growth during energy limiting conditions. We therefore propose, that blocking the bZIP regulators partly releases root meristematic growth. These findings may offer an opportunity, to further dissect how oxylipins interfere with root growth and/or meristem function.

### Expression of ERF106 and ERF107 enhances tolerance to 9-HOT

**Fig 4:**
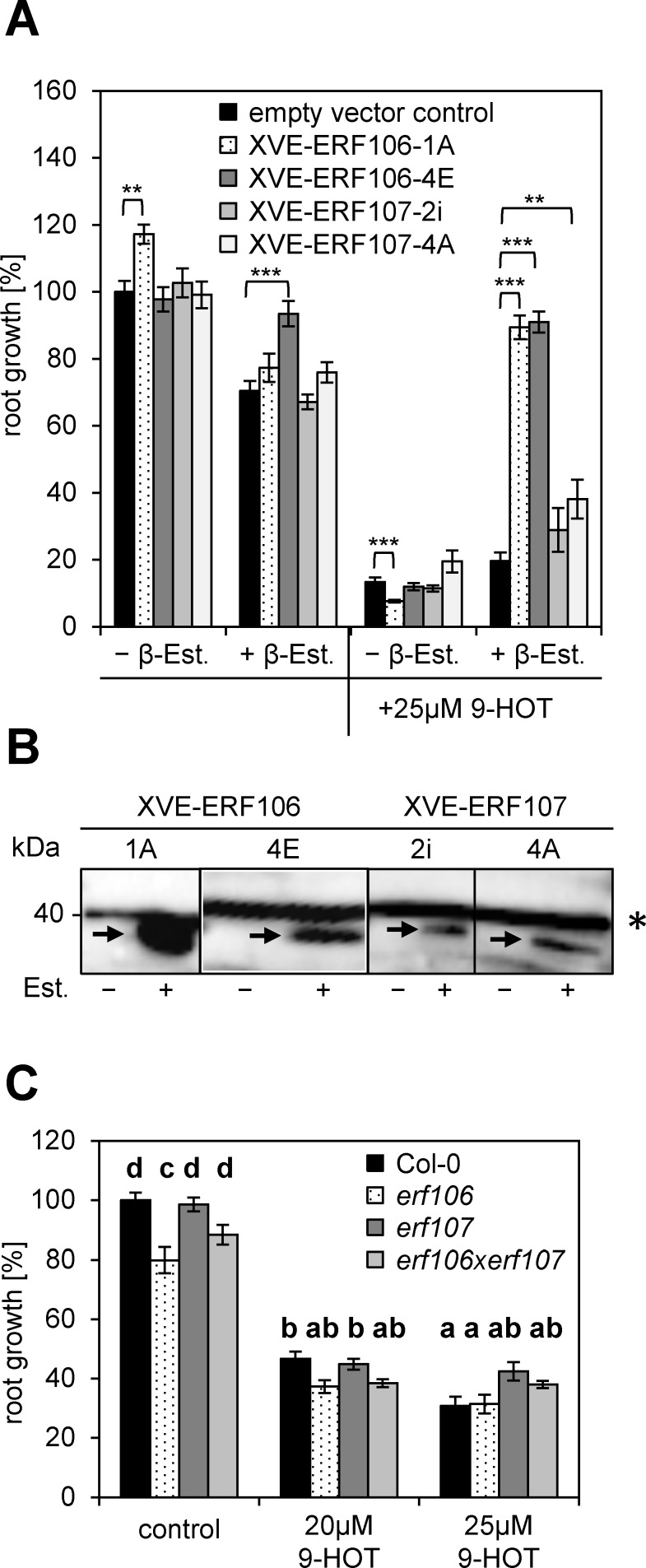
Overexpression of ERF106 and ERF107 leads to tolerance to 9-HOT. (A) Root growth of Est-inducible XVE-ERF106 and XVE-ERF107 lines was quantified after application of Est (10μM) or Est + 9-HOT (25μM). For each genotype, two independent transgenic lines are analyzed. The untreated wt (Col-0) is set to 100%. Given are mean values +SE (n≥22). The indicated differences are calculated using the student’s *t*-test: *p ≤ 0.05, **p≤0.01, ***p ≤ 0.001. (B) Immunoblot using an α-HA antibody to detect HA-ERF fusion proteins (arrows). Seedlings were grown for 5d on Medium supplemented with 10μM Est. * background signal. (C) The single and double *erf* mutants indicated show a root growth on 9-HOT which is comparable to the wt (Col-0). Significant differences between treatments are designated by different letters (one-way analysis of variance with Bonferroni post hoc test; p>0.05).

Several ERF (ETHYLENE RESPONSE FACTOR) family members from various subgroups have been identified in the *At*TORF-Ex screen (Table 1). ERF106 and ERF107, two closely related sub-group IX members [47] were found 10 times in the screen, indicating a highly reproducible function in altering the response to 9-HOT. To confirm our findings, we generated Est-inducible expression lines (XVE-ERF106 and XVE-ERF107) and tested their root growth on media supplemented with Est and/or 9-HOT. Whereas 9-HOT leads to a severe reduction of root growth, Est-induced ERF106 expression reverts growth to wt level (Fig 4A). Along this line, two independent lines expressing ERF107 show only a small, but significant increase in root growth under 9-HOT exposure. The quantitative difference may be due to varying expression levels. Immunoblot analyses detecting the ERF proteins making use of an N-terminally fused HA-tag, show lower protein levels for ERF107 compared to ERF106 (Fig 4B). As several independent XVE-ERF107 lines follow the same pattern we assume, that plants are not supporting high-level ERF107 protein expression. Indeed, the closely related ERF104 is controlled by protein stability, due to MPK6 (MITOGEN-ACTIVATED PROTEIN KINASE6) mediated phosphorylation [48].

Complementary to overexpression, we also used loss-of-function approaches. Single and double T-DNA insertion lines are characterized in S3A and S3B Fig. In accordance with data found in public expression libraries, the generally low expression of *ERF106* and *ERF107* can be induced by hypoxia or flooding. RT-qPCR studies confirmed that both lines are knock-outs (S3C Fig). Analyzing these mutants in the root growth assay on media supplemented with 9-HOT did not reveal any differences to wt plants, indicating that further genes are involved in establishment of 9-HOT tolerance (Fig 4C). The respective ERF sub-family IX [47] consists of 17 members from which 10 were found in the screen (Table 1). Presumably, overexpression of several of these ERF factors may lead to upregulation of genes involved in the tolerance mechanism. E.g. ERF104 and ERF105 have been found only once in the screen, but are highly homologous to ERF106 and ERF107. Hence, they probably share redundant functions. In transcriptome studies, performed with group IX ERF overexpression or loss-of-function lines, over-representation of genes involved in pathogen defense, stress response and/or detoxification was observed [24,49,50]. Presumably, higher-order ERF mutants are required to observe an increased sensitivity of the root to 9-HOT treatment. Redundancy between ERF102 (ERF5), ERF103 (ERF6), ERF104 and ERF5 in pathogen defense has already recently been described [50]. Importantly, transcription of *ERF106* or *ERF107* is not induced specifically by 9-HOT treatment (S4 Fig) or various pathogen stimuli [51]. However, as discussed above, post-translational regulatory mechanisms have to be taken in account.

**Fig 5:**
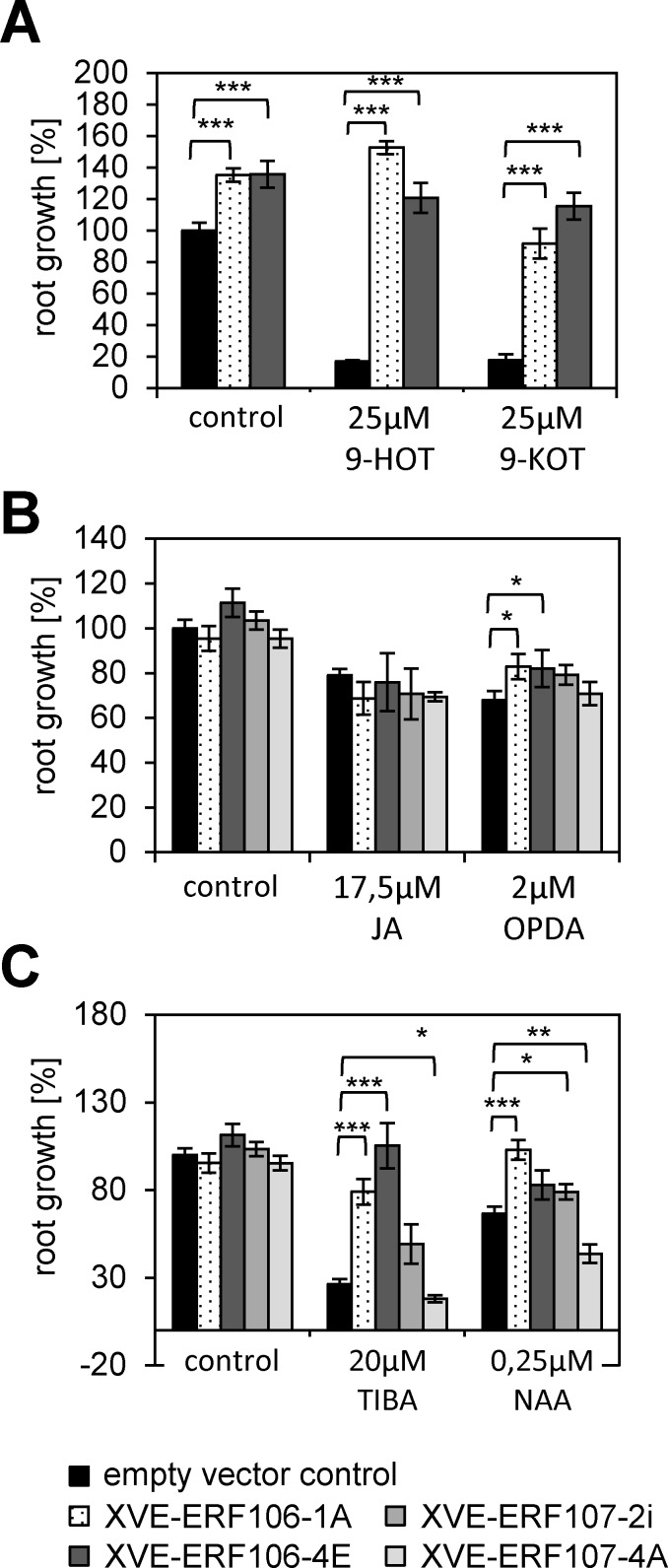
Root growth responses of XVE-ERF lines upon treatment with various oxylipin and xenobiotic compounds. Root growth of Est-induced XVE-ERF106 and ERF-107 lines treated or untreated (control) with the compounds indicated. (A) 9-LOX-oxylipins 9-HOT and 9-KOT, (B) JA and its precursor OPDA, (C) the xenobiotic compound TIBA and the synthetic auxin NAA. The untreated wt (Col-0) is set to 100%. Given are mean values +SE (n≥18). The indicated differences are calculated using the student’s *t*-test: *p ≤ 0.05, **p≤0.01, ***p ≤ 0.001.

It is conceivable, that ERF106 and ERF107 perform in general detoxification and stress responses. To analyze whether ERF106 and ERF107 are controlling specific pathways in the 9-HOT response, we tested several oxylipins, xenobiotics and plant hormones in the root growth assay. ERF106 overexpression plants showed a highly related response to the 9-LOX-derived 9-HOT and 9-KOT (Fig 5A). However, root growth in response to low levels of 13-LOX products such as JA or OPDA is clearly impaired and cannot (or only to a very minor extend) be restored by expression of ERF106 or ERF107 (Fig 5B). Moreover, overexpression of ERF106 (and to a minor extent ERF107) can restore growth inhibition by the xenobiotic compounds such as TIBA or the synthetic auxin NAA. These data support the view that particularly ERF106 induces programs to inactivate 9-LOX-oxylipins, however, displaying a broad spectrum activity to other xenobiotic or endogenously produced harmful compounds.

## Conclusions

Whereas the signaling properties of 13-LOX-oxylipins, such as JA is well-defined, the function of 9-LOX-derived oxylipins as signaling compounds is still a matter of debate. In particular, perception mechanisms are yet not elucidated. Screening approaches for components of signaling pathways are therefore straightforward [11]. Here, we can support previous findings that *At*TORF-Ex screening provides a reliable and reproducible molecular tool to identify TFs involved in stress response. Nevertheless, we could not identify TF candidates which are activating pathways specifically involved in the signaling or metabolism of 9-LOX-oxylipins. The following limitations have to be taken into account: (1) Screening for restoration of root growth during treatment with an inhibiting compound has been found to be an easy-to-use approach. However, root growth as the screening output is pleiotropic and may be caused by numerous factors, which are not specific for the oxylipin compound used. (2) It is conceivable, that oxylipin signaling is closely linked to general plant stress, detoxification or developmental processes and hence, that their responses are channeled via general transcriptional regulators.

Here, we identified two sets of TGA and ERF factors, which both induces mechanisms of detoxification/metabolism related to a broader spectrum of oxylipin and xenobiotic compounds. It will be interesting to determine, whether independent detoxification pathways are used and which endogenous substrates are detoxified *in vivo*. In animal systems, pathways leading to detoxification of xenobiotics are well-described and TF regulators are known [52]. In contrast, our knowledge on the regulation and the detoxification processes in plants is much less understood. Indeed, the TFs identified here might be a starting point for gaining insight into the plant xenobiotic responses (XR) [53]. Moreover, we identified the involvement of 9-HOT in a bZIP network which is proposed to control root growth depending on starvation conditions. Oxylipins interfere with root meristematic activity however, the precise mechanism remains elusive. Taken together, the TFs identified in this screen provide a gateway for further studies to link oxylipin signaling to cell-cycle and meristem function.

## Supporting Information

S1 Fig Root growth of *tga* mutants on 9-HOT. The *tga* triple (*tga2*,*5*,*6*) double (*tga2*,*5*) and single *tga6* mutants and the triple mutant complemented with a genomic *TGA5* fragment (*gTGA5*) [13] were assayed for root growth on 9-HOT containing medium. The untreated wt (Col-0) is set to 100%. Given are mean values +SE. The indicated statistical differences are calculated using the student’s *t*-test: *p ≤ 0.05, **p≤0.01, ***p ≤ 0.001.
(TIFF)

S2 Fig Molecular characterization of the isolated *At*TORF-Ex harboring a bZIP11 transgene. Combined PCR and sequencing analysis of three selected *At*TORF-Ex lines (A-C) revealed presence of the *bZIP11* transgene, suggesting overexpression of this TF. However, recent publications demonstrate a severe dwarf phenotype resulting from *bZIP11* ectopic expression^41^. As the *At*TORF-Ex collections are build or primary transformants, the T2 generation of the three selected bZIP11-*At*TORF-Ex lines segregates. RT-qPCR analysis of 5 offspring lines each (1–5) show no overexpression, but partially reduced *bZIP11* transcript levels, presumably due to co-suppression. Hence, these data suggest, that the observed phenotype is due to lower *bZIP11* expression.
(TIFF)

S3 Fig Molecular analysis of the *erf106* and *erf107* mutants. (A) Schematic view of the T-DNA insertions and primer attachment sites used for genotyping. (B) Homozygous mutants were identified by PCR genotyping using the primers given in S1 Table. (C) RT-qPCR analysis to verify loss *ERF* transcripts in the *erf106*, *erf 107* and double mutants. Low background transcription (black) was enhanced by 4h flooding (white). Given are mean values + SD (n≥3); *t*-test: *p≤0.05, **p≤0.01.
(TIF)

S4 Fig *ERF106* and *ERF107* transcription is not induced by 9-HOT. 2-week old *Arabidopsis* Col-0 seedlings were grown on MS-Agar supplemented with 25μM 9-HOT for 1 to 6h. *ERF106* (blue) and *ERF107* (green) transcript abundance relative to the uninduced situation was analyzed by RT-qPCR in shoot (A) and root (B). Given are mean values +SD (n≥3).
(TIF)

S1 Table Oligonucleotides used in this study. (DOCX)
