# Supplementary figures and images for: Screen Identifying Arabidopsis Transcription Factors Involved in the Response to 9-Lipoxygenase-Derived Oxylipins
Source: PLoS One. 2016 Apr 13;11(4):e0153216. doi: 10.1371/journal.pone.0153216 (PMC4830619; doi:10.1371/journal.pone.0153216)

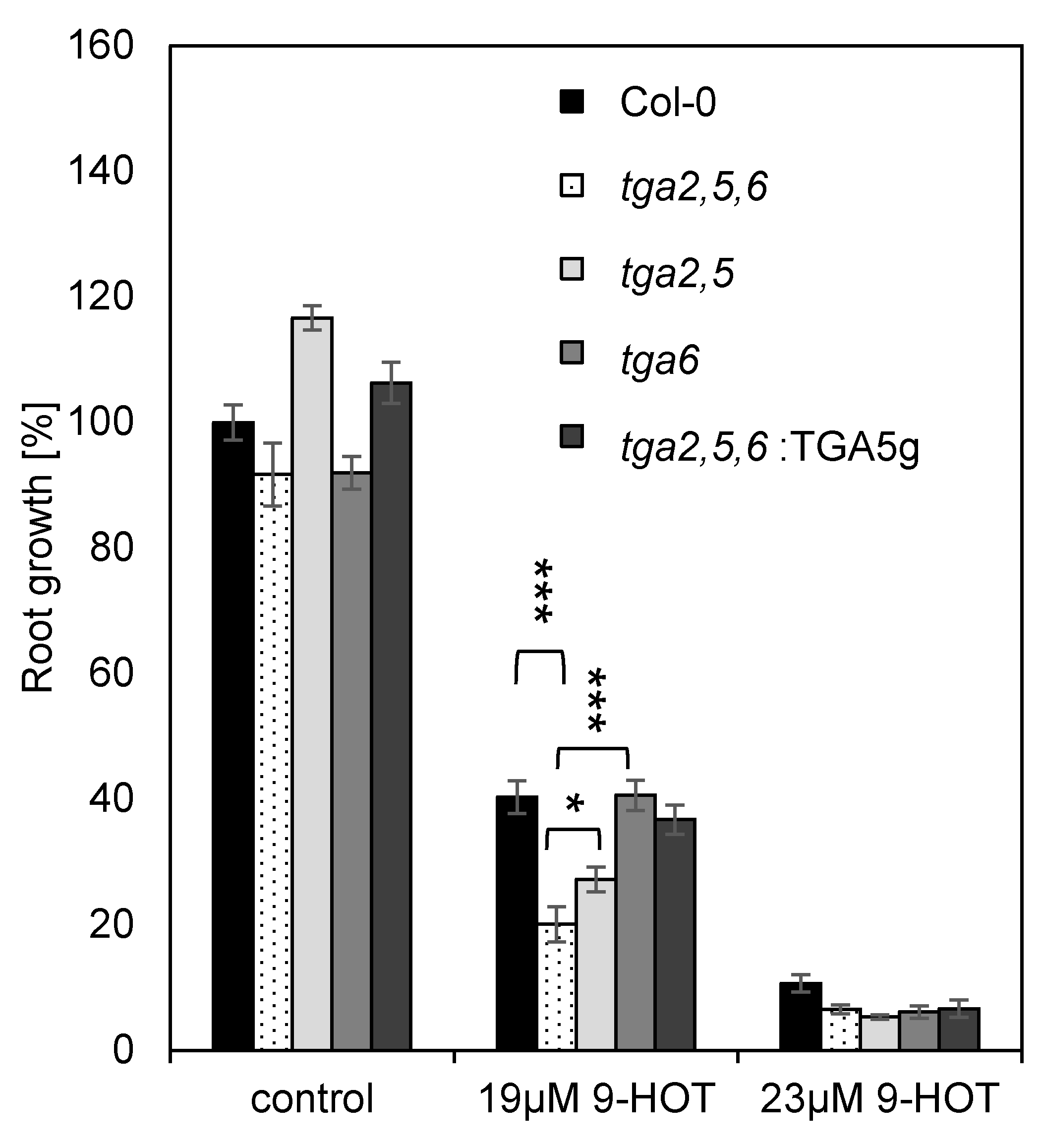

Supplement: S1 Fig — The tga triple (tga2,5,6) double (tga2,5) and single tga6 mutants and the triple mutant complemented with a genomic TGA5 fragment (gTGA5) [13] were assayed for root growth on 9-HOT containing medium. The untreated wt (Col-0) is set to 100%. Given are mean values +SE. The indicated statistical differences are calculated using the student’s t-test: *p ≤ 0.05, **p≤0.01, ***p ≤ 0.001. (TIFF) [file pone.0153216.s001.tiff]

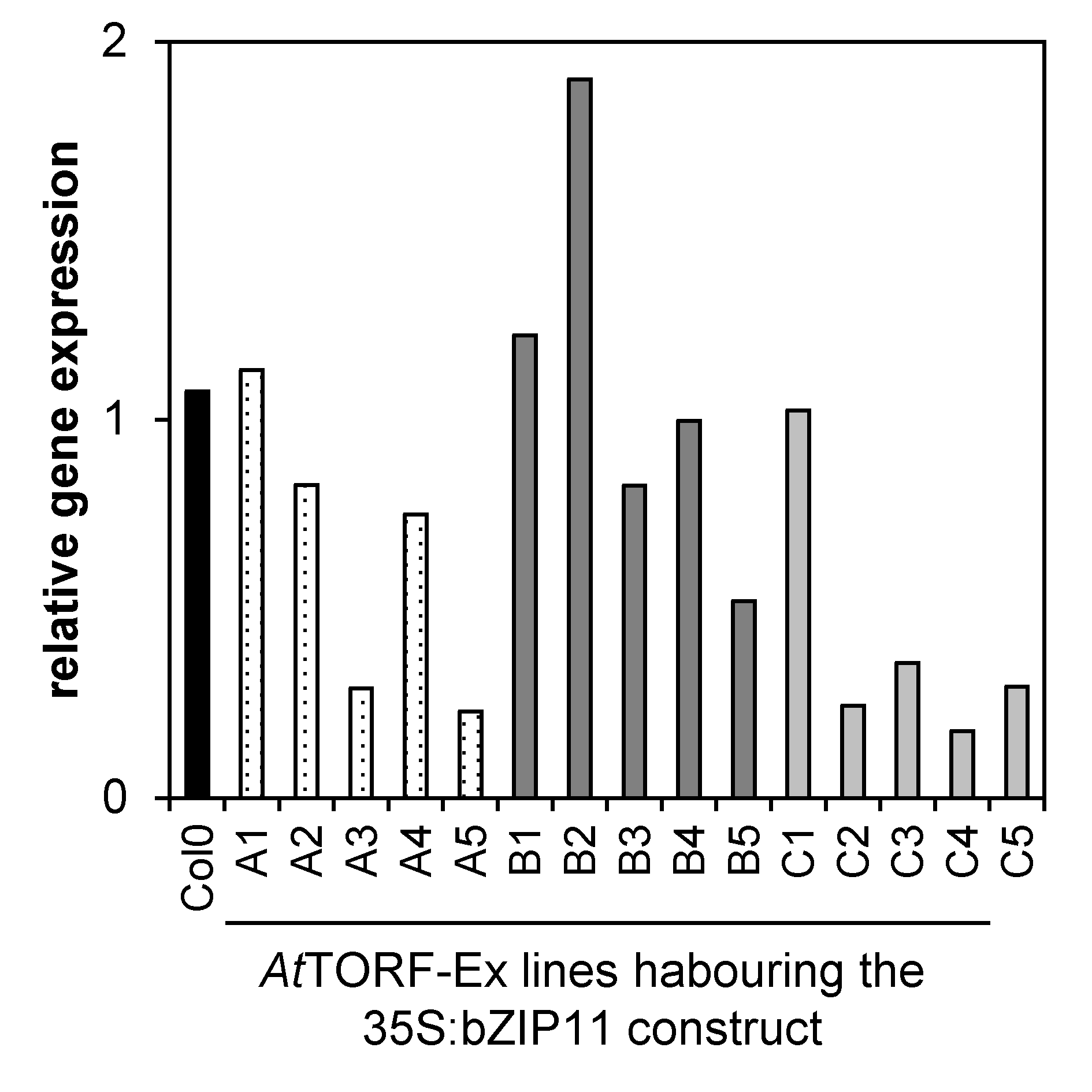

Supplement: S2 Fig — Combined PCR and sequencing analysis of three selected AtTORF-Ex lines (A-C) revealed presence of the bZIP11 transgene, suggesting overexpression of this TF. However, recent publications demonstrate a severe dwarf phenotype resulting from bZIP11 ectopic expression41. As the AtTORF-Ex collections are build or primary transformants, the T2 generation of the three selected bZIP11-AtTORF-Ex lines segregates. RT-qPCR analysis of 5 offspring lines each (1–5) show no overexpression, but partially reduced bZIP11 transcript levels, presumably due to co-suppression. Hence, these data suggest, that the observed phenotype is due to lower bZIP11 expression. (TIFF) [file pone.0153216.s002.tiff]

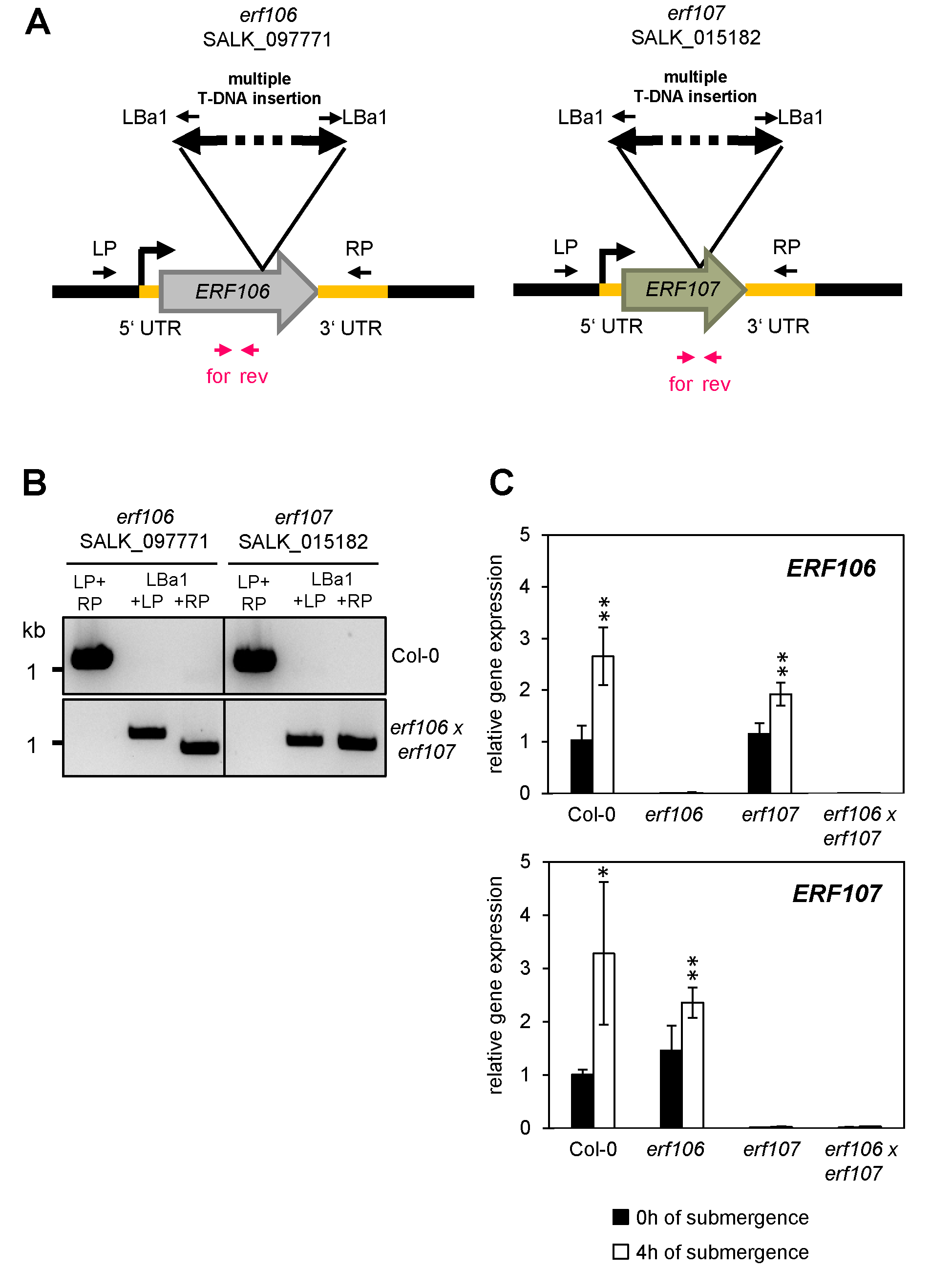

Supplement: S3 Fig — (A) Schematic view of the T-DNA insertions and primer attachment sites used for genotyping. (B) Homozygous mutants were identified by PCR genotyping using the primers given in S1 Table. (C) RT-qPCR analysis to verify loss ERF transcripts in the erf106, erf 107 and double mutants. Low background transcription (black) was enhanced by 4h flooding (white). Given are mean values + SD (n≥3); t-test: *p≤0.05, **p≤0.01. (TIF) [file pone.0153216.s003.tif]

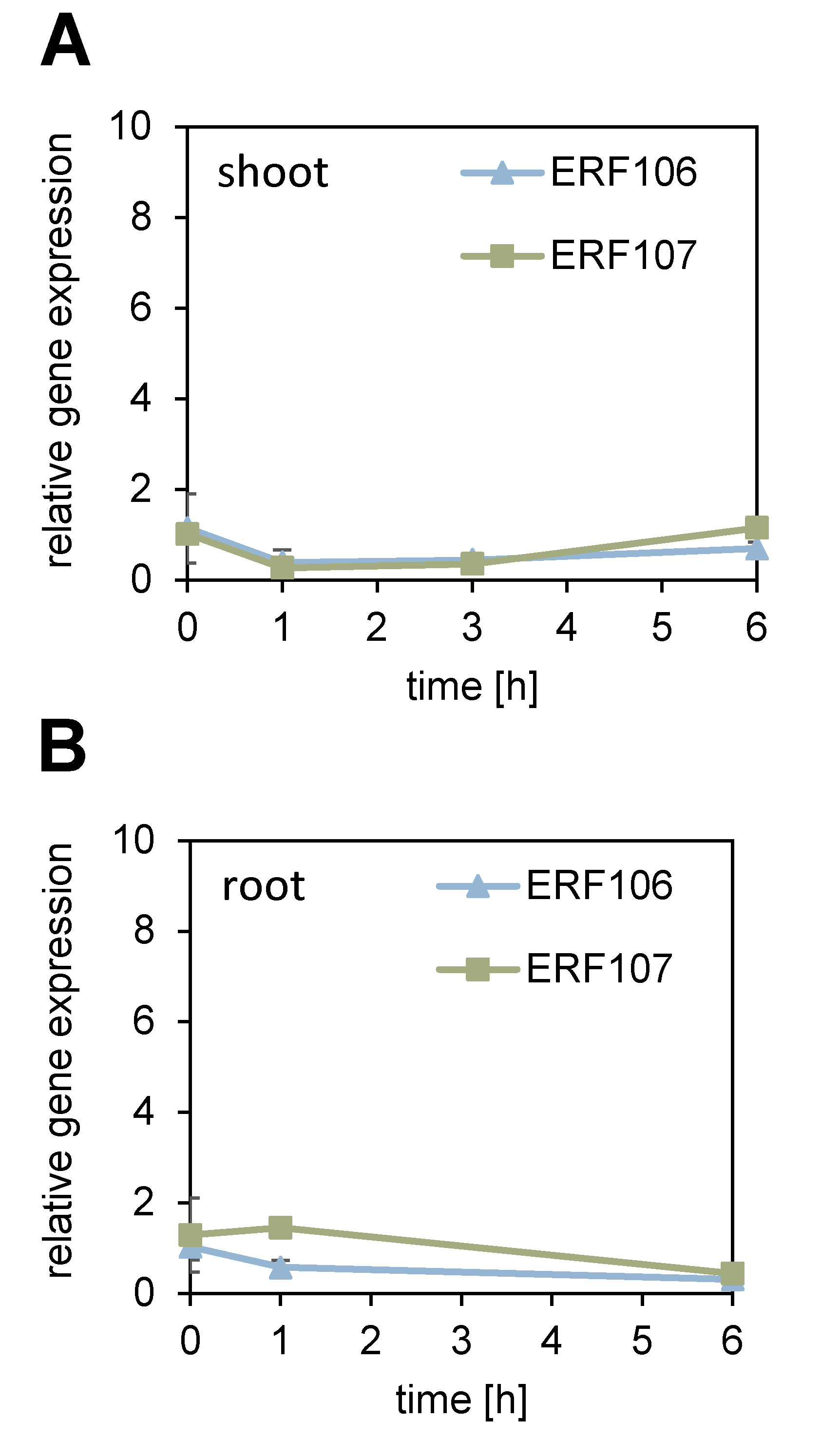

Supplement: S4 Fig — 2-week old Arabidopsis Col-0 seedlings were grown on MS-Agar supplemented with 25μM 9-HOT for 1 to 6h. ERF106 (blue) and ERF107 (green) transcript abundance relative to the uninduced situation was analyzed by RT-qPCR in shoot (A) and root (B). Given are mean values +SD (n≥3). (TIF) [file pone.0153216.s004.tif]
