# Supplementary material for: Screen Identifying Arabidopsis Transcription Factors Involved in the Response to 9-Lipoxygenase-Derived Oxylipins
Source: PLoS One. 2016 Apr 13;11(4):e0153216. doi: 10.1371/journal.pone.0153216 (PMC4830619; doi:10.1371/journal.pone.0153216)
Supplement: S1 Table — (DOCX) [file pone.0153216.s005.docx]

**Table S1: Oligonucleotides used in this study**

| **Primer name** | **Sequence** | **Experiment** |
| --- | --- | --- |
| \| attREV \| \| --- \| | CCACTTTGTACAAGAAAGCTGGG | *At*TORF-Ex |
| pE-HA-LB | CGTCCCGGACTATGCAGATA | *At*TORF-Ex |
| pE-HA-RB | CGTATGGATAACCCCCATCA | *At*TORF-Ex |
| LBa1 | TGGTTCACGTAGTGGGCCATCG | Genotyping  Salk lines |
| SALK_097771_ERF106KO_for | AGTGCCGTTGAGAATCAAATG | Genotyping  Salk lines |
| SALK_097771_ERF106KO_rev | GCAACTTGCAAGCACACTAAAC | Genotyping  Salk lines |
| ERF106 for | AAGTGGAAGGGGAATCATCA | RT-qPCR |
| ERF106 rev | AGTCTCATATGTTGATGACGATGAA | RT-qPCR |
| SALK_015182_ERF107KO_for | CAGCTTAGGATTCGAACCATG | Genotyping  Salk lines |
| SALK_015182_ERF107KO_rev | GAAAACGCAGAAGTTCCATTG | Genotyping  Salk lines |
| ERF107 for | TTTGCAGCAGAGATTCGAGA | RT-qPCR |
| ERF107 rev | AAAAGTGCCTAACCAAATCCTG | RT-qPCR |
| bZIP11 for | CGATTCAAACGTCGTCAGG | RT-qPCR |
| bZIP11 rev | TCCGTTTACGTTTCCTCTGC | RT-qPCR |
